# KRAS G12 isoforms exert influence over up-front treatments: A retrospective, multicenter, Italian analysis of the impact of first-line immune checkpoint inhibitors in an NSCLC real-life population

**DOI:** 10.3389/fonc.2022.968064

**Published:** 2022-11-14

**Authors:** Sara Fancelli, Enrico Caliman, Francesca Mazzoni, Luca Paglialunga, Marta Rita Gatta Michelet, Daniele Lavacchi, Rossana Berardi, Giulia Mentrasti, Giulio Metro, Ilaria Birocchi, Angelo Delmonte, Ilaria Priano, Camilla Eva Comin, Francesca Castiglione, Caterina Bartoli, Luca Voltolini, Serena Pillozzi, Lorenzo Antonuzzo

**Affiliations:** ^1^ Department of Experimental and Clinical Medicine, University of Florence, Florence, Italy; ^2^ Clinical Oncology Unit, Careggi University Hospital, Florence, Italy; ^3^ Medical Oncology Unit, Careggi University Hospital, Florence, Italy; ^4^ Department of Medical Oncology, Università Politecnica delle Marche, Azienda Ospedaliero Universitaria (AOU) Ospedali Riuniti di Ancona, Ancona, Italy; ^5^ Medical Oncology Unit, Santa Maria della Misericordia Hospital, Perugia, Italy; ^6^ Scientific Institute of Romagna for the Study and Treatment of Tumors (IRST) Istituto di Ricovero e Cura a Carattere Scientifico (IRCCS), Meldola, Italy; ^7^ Surgery, Histopathology and Molecular Pathology Unit, Careggi University Hospital, Florence, Italy; ^8^ Pathological Histology and Molecular Diagnostics Unit, Careggi University Hospital, Florence, Italy; ^9^ Thoracic Surgery Unit, Careggi University Hospital, Florence, Italy

**Keywords:** KRAS mutations, KRAS G12 isoforms, treatment responses, immune check-point inhibitors, PD-L1

## Abstract

**Background:**

KRAS is commonly mutated in non-small cell lung cancer (NSCLC); however, the prognostic and predictive impact of each G12 substitution has not been fully elucidated. The approval of specific G12C inhibitors has modified the idea of KRAS “undruggability”, and although the first-line standard consists of immune checkpoint inhibitors (ICIs) with or without chemotherapy, as suggested at ASCO 2022, the outcome in KRAS-mutated population is still controversial.

**Methods:**

We retrospectively described the clinical and pathological characteristics of a homogeneous G12 mutated cohort of 219 patients treated in four Italian oncologic units. We evaluated the outcome (PFS at 18 months and OS at 30 months) of those who underwent standard first-line treatment according to PD-L1 status, focusing on differences across single mutations.

**Results:**

In the study population, 47.9% of patients harbor the KRAS G12C mutation; 20.5%, G12V; 17.4%, G12D; and 8.2%, G12A. Smoking was a common behavior of patients harboring transversions and transition mutations. PD-L1 expression does not show particular distribution in the case series, although we recorded a prevalence of PD-L1 <1% in G12V (51.4%) compared to G12A (26.7%). ICIs alone was the clinician’s choice in 32.7% of patients, and the chemo-immune combination in 17.3% of patients. We described the independent prognostic role of young age (*p* = 0.007), female gender (*p* = 0.016), and an ICI-based regimen (*p* = 0.034) regardless of mutations. Overall, our data confirm the worst prognostic value of G12V mutation apart from treatment choice unlike the other major mutations (C, D, and A) that showed a favorable trend in PFS.

**Conclusions:**

KRAS G12 mutations are confirmed to have different characteristics, and the outcome is influenced by ICI first-line regimen. This study provides valuable information for further analysis in the future.

## Introduction

*RAS* genes encode for a family of small membrane-bound guanosine-triphosphate (GTP) binding proteins involved in the regulation of cell proliferation, growth, and mobility, as well as in apoptosis mechanisms through several downstream effectors. *RAS* mutations lead to protein conformation changes resulting in the perpetual activation of downstream pathways and a complete independence from the upstream signaling (1). Fifteen percent to 25% of non-small cell lung cancer (NSCLC) patients harbor Kras mutations that, in 95% of cases, rely on base substitution in exon 2 (2). According to the COSMIC database, G12C, G12V, G12D, and G12A mutations are the most common KRAS single-amino acid substitutions in lung adenocarcinoma (LUAD) (3), unlike the squamous cell histotype in which KRAS mutations are rare (3%–5%) (4, 5). The prognostic and predictive value of Kras mutations is still controversial. Although the prognosis appears to be correlated to the KRAS’ codon damage and to the setting analyzed (6–8), the predictive value did not find a precise characterization. In fact, the clinical trials evaluating the use of different agents [TKI, chemotherapy (CT), antiangiogenics, or different combinations of these] were inconclusive in the KRAS-mutated population (9–11). Recent results from the phase 2 trials CodeBreaK 100 and KRYSTAL-1 (12, 13) led to FDA approval of KRAS G12C selective inhibitors, i.e., sotorasib in May 2021 and the new drug application for the use of adagrasib in February 2022, for patients with pretreated KRAS G12C-NSCLC (14, 15). Despite the encouraging results, neither sotorasib nor adagrasib is still recommended as a first-line treatment in advanced KRAS G12C LUAD, and results in this setting are awaited from ongoing clinical trials (e.g., KRYSTAL-7, CodeBreak201, and NCT04933695). Monoclonal antibodies targeting the PD1/PD-L1 axis and CTLA-4 (e.g., nivolumab, pembrolizumab, atezolizumab, and ipilimumab) induce T-cell reactivation in several neoplasms, although their efficacy is patchy due to existing mechanisms of immunosuppression (16, 17). However, immunotherapy has gradually become relevant in KRAS-mutated patients, both because it is currently a standard first-line treatment alone or in combination with CT (18, 19), and because of its efficacy in these patients, as described in numerous experiences including the Keynote-042 subgroup analysis (20–23). Moreover, as recently reported at ASCO 2022, the use of chemo-immune checkpoint inhibitors enhances overall survival (OS) and overall response rate (ORR) in a KRAS-mutated population (24). It is noteworthy that Kras has been reported to influence the peritumor immune microenvironment and the expression of PD-L1 (25, 26), and *in vitro* evidence suggests a difference in the enhancement of antitumor immunity caused by different punctiform mutations in KRAS (27, 28).

The above-mentioned background has inspired this Italian retrospective study to directly evaluate in an unselected KRAS G12-mutated population the real impact of the use of chemo- or immunotherapies alone or in combination, as a frontline treatment according to demographic characteristics and single-amino acid substitutions.

## Materials and methods

### Study population

We enrolled all NSCLC patients with a confirmed diagnosis of LUAD, detected from January 2015 to December 2021 in four Italian Cancer Units [Clinical and Medical Oncology Units, Careggi University Hospital, Florence; Department of Medical Oncology, Università Politecnica delle Marche, AOU Ospedali Riuniti di Ancona, Ancona; Medical Oncology Unit, Santa Maria della Misericordia Hospital, Perugia; and the Scientific Institute of Romagna for the Study and Treatment of Tumors (IRST) IRCCS, Meldola]. Eligibility criteria included the following: age >18 years and available KRAS G12 mutation status regardless of the expression of PD-L1, which was not mandatory. We collected demographic data in an electronic record including age, sex, ECOG PS, smoking habits, data of death or last follow-up, disease characteristics such as KRAS mutational status, and PD-L1 status (<1%; 1%–49% and >50%) when available. Details about first-line treatment [date of first and last dose treatment, and best response achieved according to the Response Evaluation Criteria in Solid Tumors (RECIST) version 1.1] were gathered. The measured clinical outcomes were progression-free survival (PFS) evaluated at 18 months and OS at 30 months. All data were collected and analyzed anonymously; all patients signed an informed consent prior to starting treatment. This study complies with the Declaration of Helsinki rules of the World Medical Association and has been reviewed and approved by the Regional Ethics Committee for Clinical Trials of the Tuscany Region (approval No.: 20039_oss).

### Treatments

All patients underwent frontline therapy with anti-PD-L1, CT, or a combination of them. The drugs that were mainly used were the following: four to six cycles of cisplatin 75 mg/m^2^ or carboplatin area under the concentration time curve, 5 mg per milliliter per minute intravenous (IV) D1 Q3W, pemetrexed 500 mg/m^2^ IV D1 Q3W continued until the radiographically confirmed PD or toxicity, and pembrolizumab 200 mg IV D1 Q3W until the radiographically confirmed progression disease, toxicity, or the conclusion of 35 planned cycles. Pembrolizumab was administered alone or in combination with the CT regimen previously described. Few patients underwent a carboplatin-based regimen with paclitaxel 175 mg/m^2^ and bevacizumab 7.5 mg/kg IV D1 Q3W.

### KRAS mutation analysis

DNA was extracted from formalin-fixed paraffin-embedded tissue using a MagCore^®^ Genomic DNA FFPE One-Step Kit on MagCore^®^ Automated Nucleic Acid Extractor HF16Plus. Mutational analysis was performed as per local practice with the following panels: Myriapod^®^ NGS-LT 56G Onco panel on Ion Torrent Ion S5™ system, with Myriapod^®^ NGS Cancer panel DNA on Illumina MiSeq^®^ and with Myriapod^®^ Lung status on MassARRAY^®^. The analysis of the results of the NGS sequencing was carried out using Myriapod NGS Data Analysis Software and mutations were selected using the online genetic databases Clinvar and COSMIC [a minimum variant allele frequency (VAF) of 5% was applied for variant filtering].

### PD-L1 detection

From each block, 4-µm sections were cut and stained with monoclonal antibody PD-L1 (clone SP263, Ventana Medical System, Ventana, Tucson, Arizona) on an automated staining platform (Benchmark ULTRA; Ventana). An OptiView DAB IHC detection kit (Ventana) and an OptiView Amplification kit (Ventana) were used according to the manufacturer’s recommendations for visualization of the immunoreaction. Positive and negative controls were set parallel to the analyzed section. The positive control used was a tonsil section, and the negative control used was ready-to-use mouse serum with no immunization (Ventana). Partial or complete membrane staining of vital malignant cells was considered positive regardless of intensity. For each positive case, the percentage of viable stained tumor cells over total tumor cells (TPS) was used to categorize PD-L1 expression in three groups: TPS < 1% (negative), 1%–49%, and ≥50%.

### Statistical analysis

Demographic and clinical data, disease and treatment characteristics, treatment exposure, and outcomes were analyzed using descriptive statistics. Continuous variables were presented as median and range, and categorical data were presented as counts and percentages. The Kaplan–Meier analysis was used to estimate PFS and OS, and log-rank test was applied to test for statistical significance. Cox proportional hazards model analysis was used to generate point estimates of the hazard ratio (HR) and the corresponding 95% confidence interval (CI) to estimate the risk of each individual KRAS isoform with outcome. Survival distributions for specific subgroups of patients has been tested with log-rank test. A *p*-value of 0.05 or lower has been considered statistically significant According to the class of demographic/clinical variables, suitable multivariate models were constructed, consistent with the significance of each variable (significance identified through the respective *p*-values relating to the Student’s *t*-test of significance for each variable involved), as well as the possible significance of the interactions between the variables. All statistical analyses were performed using Jamovi (The Jamovi Project, 2021), and the creation of graphs and figures was carried out with R Statistical Software (v4.1.2; R Core Team 2021).

## Results

### Patients’ characteristics

**Figure 1:**
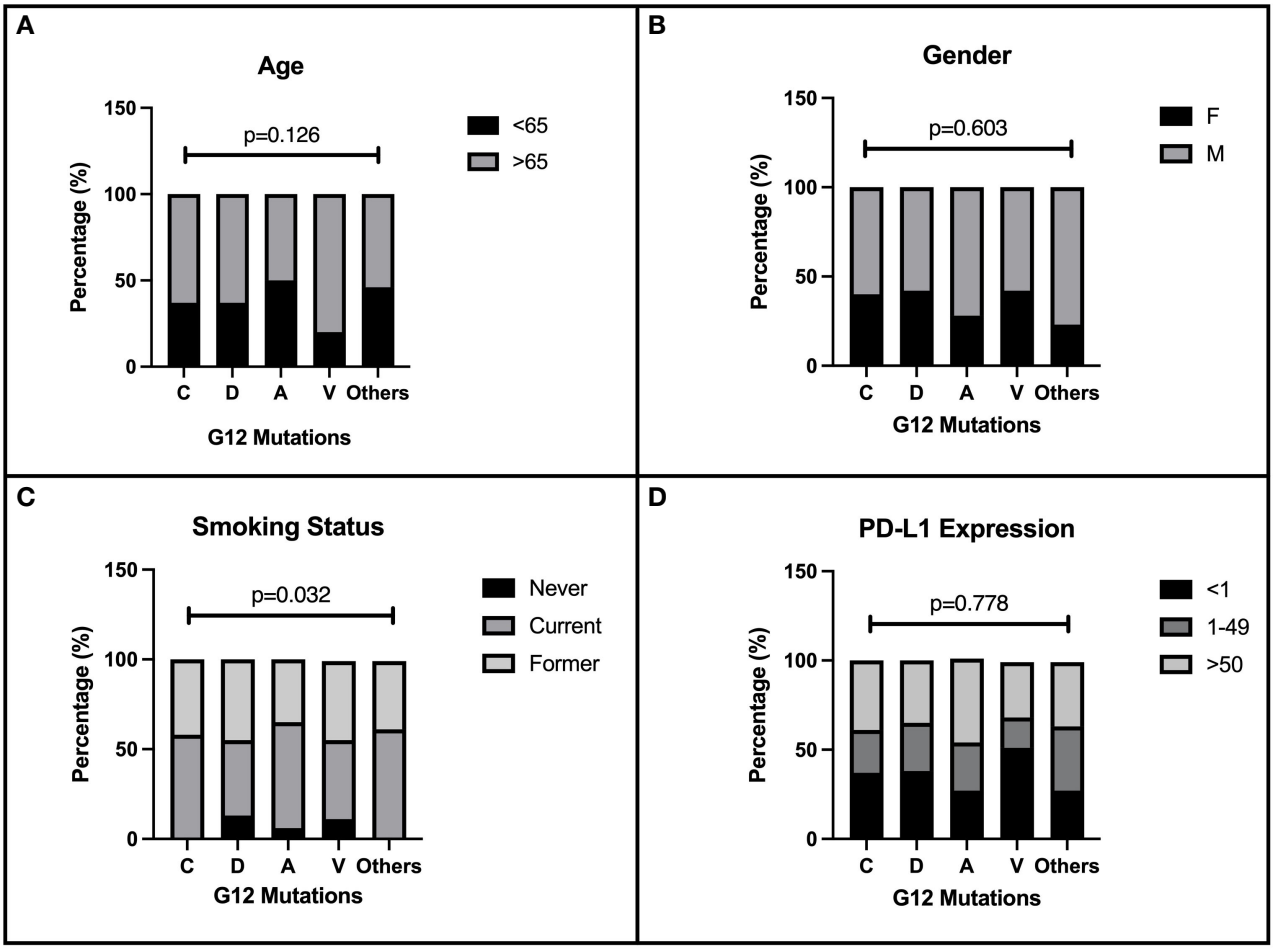
Correlation of patients’ characteristics and G12 bases. Baseline characteristics of 219 NSCLC patients enrolled in the present retrospective study. The distribution of demographic and immunohistochemical properties is shown in figure according to the single amino acid mutation. The distribution of G12 isoforms was correlated to: **(A)** age of patients at diagnosis (< 65 and ≥65 years), p=0.126. **(B)** gender p=0.603. **(C)** smoking habits p=0.032. **(D)** PD-L1 expression, p=0.778.

We evaluated 219 patients with single punctiform mutation on KRAS G12 treated in four Italian oncology units with CT or immune checkpoint inhibitors (ICIs) alone or their combination as first-line therapy for stage IV NSCLC. The baseline characteristics of the enrolled patients are described in **Table 1**. As expected, most of the patients enrolled were male (61.2%) and older than 65 years (64.8%). The majority of patients were ECOG PS 0–1 (94%). The harboring of G12 mutations was strictly related to smoking habits (*p* = 0.032). G12C, G12V, G12D, and G12A isoforms were depicted in 47.9%, 20.5%,17.4%, and 8.2% of our population, respectively, while the other isoforms (S, F, and I) were rare (lower than 6%). No particular distribution was observed regarding the expression of PD-L1 in our population. CT was the most used first-line regimen in half of the G12 mutated patients, followed by ICIs alone (32.7%). The recent (2020) approval of the chemo-immune combination in Italy has negatively influenced the sample size of this subgroup (17.3%). Data about demographic characteristics analyzed by G12 single mutation highlighted in the G12V subgroup a predominance of the elderly (80%), and an equal distribution between young (50%) and elderly (50%) in G12A. No differences in gender distribution were observed, as expected, with a prevalence of men in all mutations. However, smoking habit seems to be closely related to C isoform expression (100%), even though D and V isoforms also harbor in never-smokers (13.2% and 11.1%). We described in G12V a prevalence of PD-L1 < 1% (51.4%); conversely, G12C had 43% of PD-L1 > 50% (*p* = 0.042). Intriguingly, G12A had a higher PD-L1 > 1% expression (73.4%) than the other isoforms (**Figures 1A–D**). Even when analyzed for mutation, the most commonly used therapy was CT, unlike the chemo-immune combination (data not shown).

**Table 1:** Clinical and demographic patients characteristics.

Baseline characteristics	No. of patients (*n* = 219)
**Sex**	
Female Male	85 (38.8%) 134 (61.2%)
**Age**	
<65 ≥65	77 (35.2%) 142 (64.8%)
**ECOG PS**	
0 1 2 3	81 (37%) 125 (57%) 13 (6%) 0 (0%)
**Smoking habitus**	
Never Current Former	11 (5%) 115 (52.8%) 92 (42.2%)
**G12 mutations**	
C V D A Others^§^	105 (47.9%) 45 (20.5%) 38 (17.4%) 18 (8.2%) 13 (5.9%)
PD-L1 expression*	
<1% 1%–49% ≥50	67 (38.5%) 42 (24.1%) 65 (37.4%)
**First line†**	
CT ICI CT+ICI	104 (50%) 68 (32.7%) 36 (17.3%)

§=F, S, and I; *n = 174 patients with PD-L1 status available; †n = 208 patients treated as first line.

CT, chemotherapy; ICI, immune checkpoint inhibitor.

### G12 survival analysis

**Figure 2:**
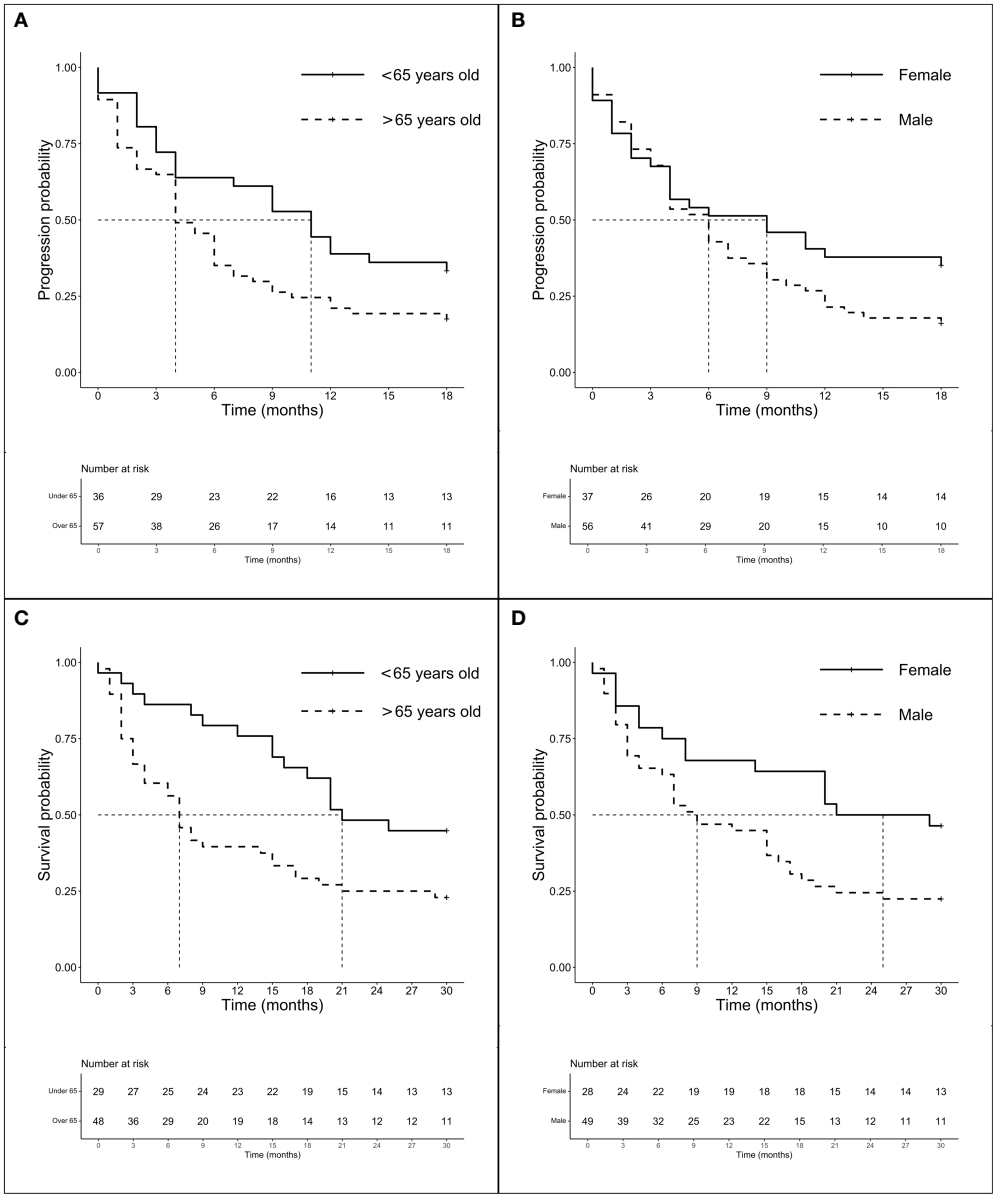
Survival analysis (PFS and OS) according to age and gender. Survival analysis (PFS and OS) of G12 patients according to age (panels A and C) and gender (panel B and D). **(A)** mPFS of patients < 65 years (*n* = 62) *vs*. patients > 65 years (*n* = 119): 6.0 months (95% CI 4.0–11.0 months) *vs*. 4.0 months (95% CI 4.0–6.0 months), *p* = 0.518. **(B)** mPFS of female (*n* = 71) *vs*. male (*n* = 110): 6.0 months (95% CI 4.0–11.0 months) *vs*. 4.0 months (95% CI 3.0–6.0 months), *p* = 0.013. **(C)** mOS of patients < 65years (*n* = 56) *vs*. patients > 65 years (*n* = 109): 20.5 months (95% CI 10.0–NR months) *vs*. 13.0 months (95% CI 9.0–17.0 months), *p* = 0.007. **(D)** mOS of female (*n* = 63) *vs*. male (*n* = 102): 23.0 months (95% CI 18.0–NR months) *vs*. 14.5 months (95% CI 9.0–17.0 months), *p* = 0.004.

We investigate the prognostic role of KRAS G12 mutations according to patients and disease characteristics and treatment chosen. Median PFS and OS of the entire population were 5.0 months (4.0–6.0 months) and 16.0 months (16.0–19.0 months), respectively, with some differences, even not statistically significant among G12 isoforms (PFS *p* = 0.518 and OS *p* = 0.593). PFS at 18 months demonstrated to be better for young people (mPFS 6.0 months, 4.0–11.0 months; HR: 1.42, 95% CI 1.01–2.0, *p* = 0.044) and for women (mPFS 6.0 months, 4.0–11.0 months; HR: 1.54, 95% CI 1.10–2.15, *p* = 0.013). The advantage was preserved for gender (*p* = 0.020) despite age (*p* = 0.150) also in multivariate analysis. No significant differences in PFS were observed according to PD-L1 expression or G12 mutation; even a trend in favor of the C isoform was found compared to V (*p* = 0.145) and for PD-L1 ≥ 50% compared to PD-L1 <1% (*p* = 0.154). The subgroup of patients exposed to ICI with or without CT showed a benefit compared to chemotherapy alone (HR: 0.63, 95% CI 0.45–0.87, *p* = 0.005) in univariate and multivariate analysis (**Table 2**) (**Figures 2A, B**). In particular, we registered a mOS of 17.0 months (13.0–29.0 months) for the C isoform (*n* = 79), a mOS of 13.5 months (6.0–26.0 months) for D (*n* = 28), a mOS of 21.0 months (7.0–NR months) for the A substitution (*n* = 13), and a mOS of 12.0 months (8.0–18.0 months) for the V substitution (*n* = 35), which proved to be the mutation with the worst prognosis. Univariate analysis upheld a better OS for <65-year-old patients (mOS 20.5 months, 19.0–NR; HR: 1.71. 95% CI 1.15–2.55, *p* = 0.008) and female patients (mOS 23.0 months, 18.0–NR; HR: 1.78. 95% CI 1.21–2.64, *p* = 0.004) (**Table 3**) (**Figures 2C, D**). The benefit of young age and female gender was confirmed also in the multivariate analysis. As per PFS, our dataset supports a trend in OS in favor of C when compared to the V isoform (*p* = 0.130) and in patients with a lack of PD-L1 expression (*p* = 0.138) when compared to patients with PD-L1 overexpression. Immunotherapy in addition or not to CT demonstrates a survival benefit in the population regardless of the G12 isoform and PD-L1 expression as shown in **Table 3** (mOS of 18.5 months, 15.0–NR; HR: 0.69. 95% CI 0.47–1.00, *p* = 0.048) benefit we also recorded in multivariate (*p* = 0.034).

**Table 2:** Univariate and multivariate progression-free survival analysis.

Characteristics	Univariate			Multivariate		
	HR	95% CI	*p*	HR	95% CI	*p*
Sex (female *vs*. male)	1.54	1.10–2.15	**0.013**	1.52	1.07–2.15	**0.020**
Age (<65 *vs*. ≥65)	1.42	1.01–2.00	**0.044**	1.30	0.01–1.85	0.150
PD-L1 (<1 *vs*. 1–49)	1.22	0.75–1.97	0.427			
PD-L1 (<1 *vs*. ≥50)	0.73	0.47–1.13	0.154			
G12 mutations (C *vs*. D)	1.05	0.66–1.67	0.830			
G12 mutations (C *vs*. A)	1.21	0.64–2.28	0.563			
G12 mutations (C *vs*. V)	1.34	0.90–2.00	0.145			
G12 mutations (C *vs*. Others)	1.40	0.70–2.81	0.343			
First line (ICI ± CT *vs*. CT)	0.63	0.45–0.87	**0.005**	0.62	0.45–0.87	**0.005**

Bold was used to pin point data with relevance.

**Table 3:** Univariate and multivariate overall survival analysis.

Characteristics	Univariate			Multivariate		
	HR	95% CI	*p*	HR	95% CI	*p*
Sex (female *vs*. male)	1.78	1.21–2.64	**0.004**	1.71	1.16–2.53	**0.007**
Age (<65 *vs*. ≥65)	1.71	1.15–2.55	**0.008**	1.63	1.09–2.44	**0.016**
PD-L1 (<1 *vs*. 1–49)	1.12	0.61–2.06	0.707			
PD-L1 (<1 *vs*. ≥50)	1.47	0.88–2.44	0.138			
G12 mutations (C *vs*. D)	1.38	0.84–2–27	0.209			
G12 mutations (C *vs*. A)	1.13	0.57–2.23	0.725			
G12 mutations (C *vs*. V)	1.44	0.90–2.29	0.130			
G12 mutations (C *vs*. Others)	1.41	0.67–2.98	0.365			
First line (ICI ± CT *vs*. CT)	0.69	0.47–1.00	**0.048**	0.66	0.46–0.97	**0.034**

Bold was used to pin point data with relevance.

### Treatment survival in G12 amino acid substitutions

**Figure 3:**
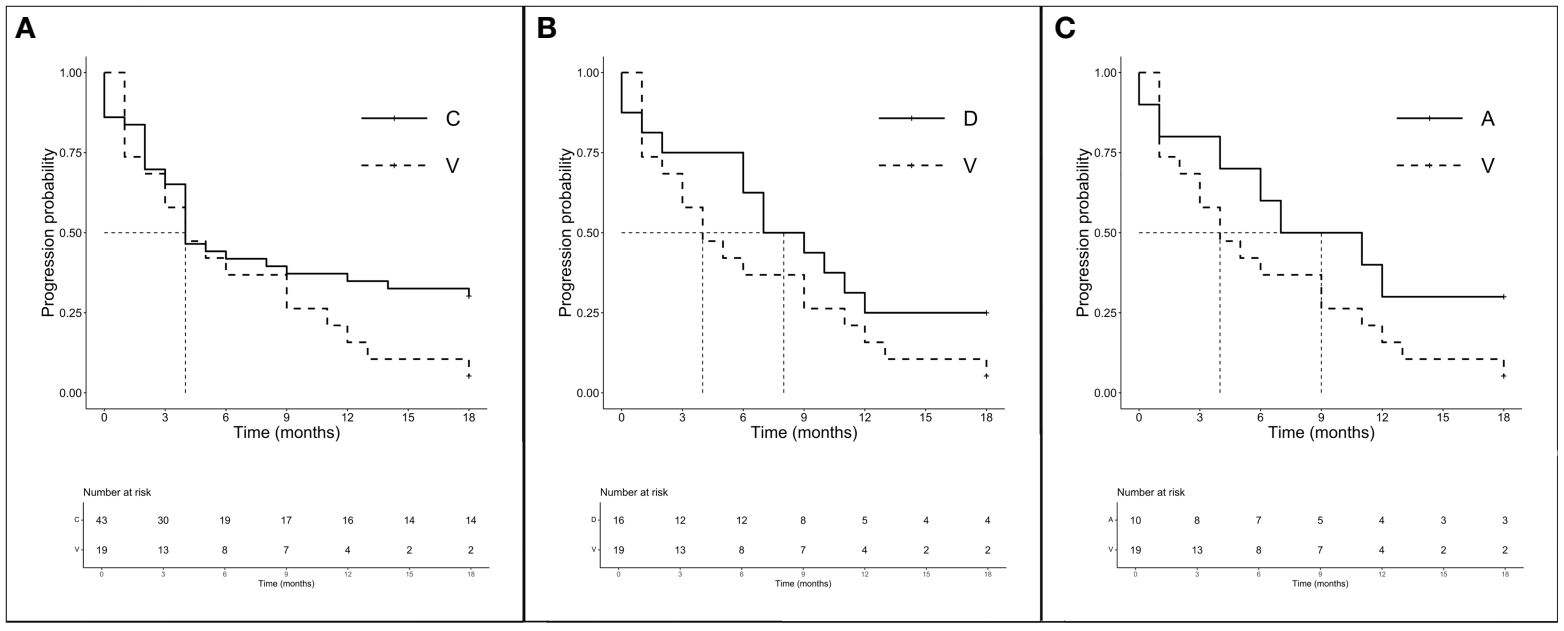
PFS according to single amino acid substitutions in patients treated with ICI ± CT (*n* = 94). **(A)** mPFS C (*n* = 43) *vs*. V (*n* = 19): 4.0 months (95% CI 4.0–18.0 months) *vs*. 4.0 months (95% CI 3.0–12.0 months), *p* = 0.114. **(B)** mPFS D (*n* = 16) *vs*. V (*n* = 16): 8.0 months (95% CI 6.0–NR months) *vs*. 4.0 months (95% CI 3.0–12.0 months), *p* = 0.165. **(C)** mPFS A (*n* = 10) *vs*. V (*n* = 16): 9.0 months (95% CI 4.0–NR months) *vs*. 4.0 months (95% CI 3.0–12.0 months), *p* = 0.140.

In accordance with previous results, we decided to analyze the amino acid substitutions’ outcomes according to treatments received. Among 106 CT-treated patients, global mPFS was 6.0 months (3.0–6.0 months) and no significant differences in mPFS were observed between the major mutations (A, C, D, and V) ranging from 3.5 to 4 months (*p* = 0.591). The mOS was 16.0 months (12.0–19.0 months) and none of the main isoforms analyzed seems to benefit from CT (*p* = 0.800). We then evaluated the outcome of treatment including ICI both on the total population (*n* = 94) and according to a major single mutation. Intriguingly, although the global mPFS was 6.0 months, isoforms C and V showed the same lower median of 4.0 months (3.0–12.0 months for V and 4.0–18.0 months for C). Mutations D and A instead showed higher mPFS (8.0 months, 6.0–NR and 9.0 months, 4.0–NR, respectively). Later, we chose to compare the outcomes of the better prognosis mutations C (*n* = 43), D (*n* = 16), and A (*n* = 10) with isoform V (*n* = 19) to evaluate the importance of the individual mutations in a population homogeneously exposed to ICI (**Figures 3A–C**). Interestingly, despite mPFS being similar in the two groups, we highlighted a favorable trend in PFS for C compared with the V isoform (*p* = 0.114) (**Figure 3A**). As shown in the figure, a similar trend was also described for D (*p* = 0.165) and A (*p* = 0.140) mutations when compared to V (**Figures 3B, C**). The mOS of the ICI-exposed subgroup (*n* = 78) was 15 months (8.0–20.0 months). As seen for PFS, V proved to be the mutation with the worst prognosis (mOS: 9.0 months, 4.0–NR) unlike the C (15.0 months, 6.0–NR), D (11.5 months, 7.0–NR), and A (20.0 months, 15.0–NR) mutations even if the comparison between better-survival mutations and the V isoform showed no significant differences. Finally, the PD-L1 expression seems to not have a prognostic role. We observed the worst outcome for those expressing PD-L1 > 50% (mOS 8.50 months, 4.0–21.0 months) who were exposed to ICI alone, while patients with PD-L1 <1% or 1%–49% who underwent CT-ICI had a mOS of 17.0 and 15.5 months, respectively (*p* = 0.404).

## Discussion

The role and characteristics of intracellular membrane proteins from the RAS family as the hub for signaling of receptor tyrosine kinases (RTK) (29, 30), G-protein-coupled receptors (GPCR) (31), and members of the integrin family (32) have been known for decades. The switch between an inactive GDP-bound and an active GTP-bound state, mediated by GTPase activating protein (GAP) and guanine nucleotide exchange factors (GEFs) (33, 34), was a well-known mechanism leading to several downstream pathways’ activation, especially RAF1 and PI3K (1). Somatic Ras mutations are prone to different switching times between an active and an inactive Ras state and to different GTP hydrolysis rates (27, 35), and the activation of different downstream pathways according to different single-amino acid substitutions in mutant KRAS tumors has been described (36–38). This evidence supports the renowned undruggability of KRAS directly or through several targets of up- or downstream signaling.

The recent discovery of drugs (sotorasib and adagrasib) able to selectively bind G12C with favorable efficacy/toxicity ratio has modified the concept of KRAS inhibition (39), and we are eager to know the results regarding their efficacy and clinical outcome (NCT04303780; NCT04685135). Despite the efforts and encouraging results of phase 2 trials in the post-first-line setting (14, 15), sotorasib and adagrasib are not recommended as frontline treatment in advanced KRAS G12C LUAD, and the final results of ongoing first-line clinical trials are still pending (e.g., KRYSTAL-7, CodeBreak201, and NCT04933695). Moreover, results on drugs targeting other mutations are still lacking even if several promising drug candidates emerge as inhibitors of other KRAS mutants as per EX185 designated to inhibit KRAS G12D, G12C, and G12V and to engage GNP-bound KRAS (40) or MRTX1133, which has shown efficacy in the KRAS G12D mutant xenograft mouse tumor model (41). Immunotherapy in combination or not with CT according to PD-L1 expression is still the standard in this wide population (18, 19, 42).

In our cohort, we analyzed a homogeneous population of KRAS G12-mutated LUAD patients (*n* = 219) undergoing first-line therapy with CT or ICI alone or their combination. The aim of the study was to characterize a possible unique profile to discriminate each single G12 mutation by treatment outcome or demographic characteristics. According to data from the COSMIC database, we observed comparable G12 amino acid substitution percentage in our population; in particular, the major detected substitutions were C (47.9%), V (20.5%), D (17.4%), and A (8.2%) (3), while the other isoforms (e.g., G, F, S, and I) were rare (less than 6%).

In accordance with Riely and colleagues (43) and preclinical evidence supporting the development of KRAS mutations due to epigenetic changes after tobacco exposure (44), we confirmed the prevalence of the KRAS G12 mutation in patients with smoking habit (95%), especially in transversions (G → T/C). Different from literature, we also described in transition mutations (G → A) a predominance of smokers (86.8%). Patients enrolled in our analysis were predominantly elderly (64.8%) and male (61.2%), which is comparable to the global LUAD distribution. Age and gender results are associated with outcome: female gender was an independent prognostic factor for longer PFS and OS and age <65 years correlated with better OS.

The different sensitivity of the specific mutant KRAS to different treatments (CT, TKIs, or anti-angiogenetics) has been widely described (45, 46), and previous *in vitro* and clinical data suggest that different KRAS mutations have different depths and durations of response to old-fashioned treatments (36, 47, 48). In addition, CT agents as platinum-derived drugs and the anti-metabolite pemetrexed have immunomodulatory properties, being able to increase MHC-I expression and recruitment of effectors (e.g., TILs, macrophages, and memory T cells) and to reduce the activity of players of the immunosuppressive microenvironment such as Tregs (49, 50). Nevertheless, in our analysis, the use of CT was globally unsatisfactory and none of the mutations analyzed had a solid benefit either in PFS or in OS. Based on the results, we investigated the efficacy of ICIs alone or in combination in our population, finding a 38% decreasing risk of disease progression and 34% death in patients receiving first-line treatment including ICI with or without CT.

Several studies attempted to determine the impact of KRAS mutations on the outcome of patients treated with ICI in the advanced setting with contradictory results (51–54). The gain from the addition of ICIs in KRAS-mutated patients is consistent with several retrospective analyses in real life (20–22) and supported by the results of the analysis of the mutated KRAS subgroup in the Keynote-042 trial, where pembrolizumab instead of CT had an ORR of 56.7% versus 18%, and similar benefits were registered in the G12C subgroup with PFS and OS similar to the entire population enrolled (23). Recently, at ASCO 2022, Nakajima and colleagues described an improved OS and ORR in a KRAS-mutated population treated with chemo-immune combination as frontline treatment, confirming our observations (24).

It should be noted that the outcome of KRAS-mutant treatments is dominated by co-occurring genetic events, as STK11/LKB1 and KEAP1 mutations define a subset of “cold” NSCLC resistant to ICI, while *TP53* alterations increase the inflammatory microenvironment, leading to an efficient immune response. However, clinical reports and retrospective gene sequencing did not identify a specific association between certain comutations and single KRAS isoforms (55, 56). Furthermore, the use of gene panels including these comutations has a patchy distribution in clinical practice; thus, information regarding this in our retrospective case series is missing.

The PD-1/PD-L1 axis is involved in the inhibition of the immune system and more specifically in the self-tolerance and regulation of T lymphocyte activity (57). Chen and colleagues described *in vitro* the ability of KRAS to upregulate PD-L1 *via* p-ERK, inducing apoptosis of CD3+ T cells and resulting in immune escape, an unfavorable condition that can be reversed by anti-PD-1 antibody (25). The involvement of other signaling pathways supporting the expression of PD-L1 as MAPK, together with STAT3, but not PI3K, has also been suggested (58). Later, the role of the Akt-mTOR axis (59) and PD-L1 miRNA stabilization (60) offered further evidence of the complexity of the influence of KRAS to PD-L1 in NSCLC. Falk and colleagues (61) described a better prognosis in patients with PD-L1 TPS ≥ 1% in KRAS*^mut^* than in KRAS*^wt^* exposed to ICI, and several other clinical retrospective examples and literature review support Falk’s observations (52, 54, 62). It is evident that the KRAS mutational status could be a potential biomarker of favorable outcome for ICI treatments; however, pivotal ICI trials did not provide univocal data on either the efficacy of these treatments or the biomarker role of PD-L1. In contrast to Falk, our data suggest a trend towards a better prognosis for patients with PD-L1 <50%. These results were partially confirmed in the subgroup analysis of patients treated with ICI with/without CT where an absence or lower PD-L1 expression seems more advantageous than PD-L1 ≥50%. According to the Italian Medicine Agency, PD-L1 TPS ≥50% is the requirement to prescribe pembrolizumab while the combination with CT is allowed only in patients with expression of PD-L1 <50%, and this could explain the benefit of the population with PD-L1 <50% seen in our analysis. Despite the promising preclinical observations, pivotal clinical trials with the ambition to demonstrate the superiority of CT-ICI combinations did not allow the enrollment of patients with KRAS mutations; therefore, there is a lack of concrete efficacy results in this population.

Even more complex is the understanding of the predictive value of response to treatments when considering individual mutations. We have previously remarked on the lack of benefit across isoforms from the use of CT, which is consistent with the observation by Wiesweg and colleagues who described that isoforms have the same intermediate prognosis (48). Thereafter, we explored the prognostic value of G12C, V, D, and A undergoing ICI ± CT and we instead noted a more unfavorable trend in PFS for G12V compared to G12C, and although mOS between mutations was almost doubled for C (9.0 months *vs*. 15.0 months), the difference was not relevant. Furthermore, V has a worse prognosis than the remaining isoforms, particularly A and D. Differences in OS are observed in these subgroups, although not statistically significant. Ihle in 2012 (36) described a poor outcome affecting patients in the BATTLE-1 trial with isoforms C and V mutated, probably due to the differences in pathway activation, which are supported by *in vitro* analysis highlighting a predominance of p-Akt activation in the D isoform, and a predominance of RalA/B for C and V isoforms. In the BATTLE-1 trial, patients were exposed to several TKIs; however, this evidence suggested a modulation of the pathways when mutations are exposed to similar treatments. Similar to our data, the mPFS of C and V isoforms exposed to ICI was 4 months, but the authors highlighted a worsening PFS in those who have *STK11/LKB1* comutations (63); unfortunately, this information is not available in our dataset. The disadvantageous weight of the V isoform has been extensively described (47, 64–66), and contrary to what has been reported in the literature (63, 67), our series was released from the high expression of PD-L1. As a matter of fact, in our case series and different from literature, the A isoform has a better outcome to ICI treatments, and this is the mutation with a higher rate of PD-L1 expression (>1%: 73.4%) and young patients, while the V mutation shows its worst prognosis in the elderly. Those results are in conflict with the study by Shen Mo et al. (68), according to which KRAS G12D and G12V mutations are better candidates for immunotherapy, whereas patients with KRAS G12A or G12C mutations are not. Intriguingly, and the same as what Jeanson and colleagues described (62), we noticed a better prognosis in those mutations as per A, which expressed a high rate of PD-L1, even if it is not possible to draw final conclusions given the small number of the subgroup.

## Conclusions

Despite the limitations derived from the retrospective characteristics of the study and the relative lack of comutation assessment, we can affirm that, to our knowledge, this is the first multicenter, real-life study with this sample size aimed only at G12 mutations in first-line patients undergoing treatment including ICI. We confirmed the scarce efficacy of CT alone in this population, which instead benefits from the use of ICI alone or in combination with CT, a benefit not linked to PD-L1 overexpression. We also confirmed the benefit in some isoforms (C, D, and A) and the negative prognostic value of the V mutation, which maintains a poor prognosis regardless of the treatment chosen, probably related to an aged population and the relative lack of PD-L1 expression in the subgroup.

## Data availability statement

The original contributions presented in the study are included in the article, further inquiries can be directed to the corresponding author.

## Ethics statement

The studies involving human participants were reviewed and approved by Ethics Committee of Area Vasta Centro (CEAVC) part of the Regional Ethics Committee of the Tuscany Region. The patients/participants provided their written informed consent to participate in this study.

## Author contributions

Conceptualization: SF, EC, and SP. Collection of data: SF, GMen, IB, MM, GMet, IP, and CB. Interpretation of data: SF, SP, and EC. Data curation: LV, CC, FC, and CB. Writing—original draft preparation: SF. Writing—review and editing: FM, SP, LA, and LP. Supervision: AD, RB, and LA. All authors have read and agreed to the published version of the manuscript. All authors contributed to the article and approved the submitted version.

## Funding

The data collection and the writing of the entire project are partially supported by ERA PerMed, having participated in the call “Second Joint Transnational Call for Proposals (JTC) 2019 on Personalized Medicine: Multidisciplinary Research Towards Implementation within the framework of ERA PerMed”, passed the final selection, and obtained the required funding and funds of Professor Antonuzzo, Department of Experimental and Clinical Medicine, University of Florence.

## Conflict of interest

The authors declare that the research was conducted in the absence of commercial or financial relationships that could be construed as a potential conflict of interest.

## Publisher’s note

All claims expressed in this article are solely those of the authors and do not necessarily represent those of their affiliated organizations, or those of the publisher, the editors and the reviewers. Any product that may be evaluated in this article, or claim that may be made by its manufacturer, is not guaranteed or endorsed by the publisher.
